# Chronic Catarrh, Pharyngitis, Laryngitis, Bronchitis, Etc.

**Published:** 1869-04

**Authors:** Wm. M. Cornell

**Affiliations:** Boston


ART. II.—*Chronic Catarrh, Pharyngitis, Laryngitis, Bronchitis, etc.* BY WM. M. CORNELL, M. D., LL. D., *of Boston.*

Catarrh is a very common difficulty, usually couched under the name of a *bad cold;* and, indeed, we never knew any one to have a *good* cold. In many cases this passes off in a few days and leaves the general health unimpaired. This, however, is not always the case. Often, through neglect or inattention, the difficulty becomes *chronic*, and is not troublesome only, but leads on to serious disease. The symptoms of this disease are more or less pain in the head, usually deep seated, chiefly in the forehead; the nostrils are dry, with a kind of burning sensation in them; the eyes are often filled with tears; there is frequent sneezing, with some fever. These are all common at the commencement of a catarrh.

Few persons understand how cold is taken. If this point were well known, there would not be so many colds. People bundle up, when they go out, and this is well enough, if they are to ride or sit still; but very poor policy if they are to walk. It is not often that colds are taken when persons are out. They are generally taken when they *come in.* When out, the cool air, so far as it comes in contact with the respiratory organs, drives the blood from them; and, when they come to the fire, or from the low temperature out of doors, into a heated room, there is a rush of blood into these vessels which very much resembles the crackling of dry thorns when they burn. It is then that cold is taken. This may be avoided by coming slowly or by degrees into a heated atmosphere. But we rush in haste to the fire, or into a room heated to seventy degrees by a detestable furnace, which ought always to come under the "Nuisance Act" in Massachusetts.

We have stated how cold is taken, because the old adage "an ounce of prevention is worth a pound of cure," is but too true.

After the first symptoms above described, a discharge of a thin, watery fluid takes place, of an acrid character, which irritates the parts over which it flows. Soon this changes from the clear and watery character and assumes a thick, yellowish color. This is the usual course of a *common cold*, or catarrh. The disease, however, does not always subside in this way, nor does it always terminate so favorably. On the contrary, the "dropping from the head" of this acrid matter excoriates the parts below, upon which it falls—

the fauces, uvula, or soft palate, the tonsils, the trachea, or windpipe, and, finally, the bronchial tubes and the lungs. Of course, the tendency is downwards.

Here, again, under proper treatment, these symptoms soon disappear. But in most cases there is no treatment at all. It is not very troublesome at first, and the person lets it alone—lets it take its course, and that course is often from bad to worse. He feels some inconvenience; has a dull pain in the forepart of the head, a little soreness about the upper part of the throat, a constant tendency to cough, and spits up every morning a few mouthfuls of a thick, greenish, sticky phlegm. Thus he goes on for weeks, perhaps months; we have seen cases where this work of destruction has been going on for years.

After the first described symptoms have changed to the second stage the thin, watery discharge having become thick, greenish and tough, the nose and passages above in the forepart of the head, begin to fill up, the senses of smell, taste and hearing become impaired. Then comes on the discharge from the throat of the thick, green, tough substance spoken of above. This is usually called the *third* stage of the disease. In many cases the discharge now becomes very offensive, in some instances so much so that one can scarcely stay in the room with the patient.

In these worst cases, there is usually a *scrofulous* habit, and remedies must be addressed, not to the diseased parts only, as they appear, but also to the general system. Persons subject to chronic catarrh are almost always taking cold, and we seldom see them when they are not "pretty well, only they have taken a little cold." It is a fact that they do take cold readily, on account of the sensitive state of the mucous or lining membrane of the respiratory organs.

After chronic catarrh has made some progress, or, indeed, as soon as it has become *chronic*, there is usually a little hacking cough, and a great deal of *clearing up* of the throat. There is a continual irritation kept up by the falling of the material already described, from the head, and hence this disease goes on from bad to worse. Soon the larynx, or vocal cords, become more or less diseased, being enlarged, inflamed, or ulcerated. The voice then grows hoarse or husky, and the more it is used the worse it feels, and the worse it *is*.

Twenty or twenty-five years ago these *throat diseases* were complained of chiefly by clergymen and singers, but in later years other classes have been attacked with the same troubles, and we now find cases among those who never sing and never speak in public. These arise chiefly from *chronic catarrh;* and persons are as liable to have this, who never use their vocal organs to any great extent, as those who do. It arises from various causes, chiefly a scrofulous constitution or impoverished blood, or some hereditary taint.

A few years ago this disease usually terminated in tracheal or bronchial consumption, as it does now when proper remedies are not applied. But at the present time facilities for treating this disease have been greatly multiplied, and if seasonably applied, often effect a cure. The chief improvement consists in conveying remedies directly to the parts diseased by atomized inhalation.

When Dr. Greene of New York, proclaimed that he could enter the larynx and trachea with a probang or crooked bone and a piece of sponge saturated with some medicated liquid, nearly all the physicians of that city protested that it could not be done, and now to read what they then wrote, though but a quarter of a century ago, reminds one of the persecution of Jenner and of Harvey, by their medical brethren. Greene, however, lived long enough to see his discovery triumphant, which neither Jenner nor Harvey did.

At the present time we have advanced far beyond anything that Greene thought of in diagnosticating diseases of the respiratory organs. Physicians can actually *do* what clairvoyants have long *pretended* to do, to wit: *look into the human body.*

The *laryngoscope!* what's in a name? especially a jaw-breaking one—is a small, round glass, that may be placed in the back part of the mouth, mounted upon a wire, at an angle of forty-five degrees, held by its handle. This comprises the sum total of this instrument. By placing it properly in the back part of the mouth, the reflecting surface shows the inside of the laryngeal box, and the vocal cords in their natural position, and the manner of their action. Even the *chink* of the glotis, as it is called, is laid open to inspection by the naked eye. This is a wonderful improvement in our pathological investigation of these parts. The precise point of disease being thus made visible, a surgical operation may readily be performed, if necessary, or atomized remedies carried to the very spot on which disease has fastened.

To a child with true croup, but before the false membrane had made much progress, I administered the spray of *lime water*, of the ordinary strength, atomized. In a very few minutes the membrane became softened and came off in small flakes. The child recovered. Medicine was also given.

To a lady with diphtheria, *lime water* of the same strength and in the same manner as in the case of the child, was employed. The lady recovered. Medicine was also given.

A lady with chronic bronchitis, accompanied with very offensive secretions, inhaled *tar water* two drachms, ordinary strength. The secretions, after a few inhalations diminished, and she recovered. A patient with gangrene of the lungs inhaled *tar water* as in case third, with a very good result. In bronchitis, *turpentine* was inhaled with good success, from two to three drops at a time. In asthma, with considerable nervous excitement, the *arsenated liquor potassa*, ten drops, was inhaled with benefit. In a vexing irritative cough, the *watery extract of opium* was inhaled, one-half a grain, with advantage. Whooping-cough in one patient, relief was afforded by the first inhalation of eight drops of the fluid extract of *hyoscyamus.* Spasmodic cough was relieved by inhaling one grain of the extract. Relief was given in consumption by inhaling from five to ten drops of the tincture of *Cannabis Indica.*

A patient with catarrh was relieved by inhaling *muriate of ammonia* up the nasal passages, fifteen grains. In my own case, benefit was derived from inhaling this remedy in bronchial cough and laryngeal irritation, which I reported some time since in the *Buffalo Medical Journal.* I have also used liquor soda chlorinate, or *chlorinated soda*, one-half to one drachm in consumption and in bronchitis with advantage.

Aphonia, caused by chronic laryngitis, was cured by a few inhalations of *sulphate of zinc*, from two to five grains at a time. Ulcerated larynx and enlarged and irritable vocal chords were greatly benefited by inhalations of *nitrate of silver;* eight grains to the ounce of water will usually be found about the proper dose; the face should be covered.

The following case of consumption were relieved very much:

A young man, right lung diseased; various preparations of iron were tried, but the best result was obtained from the *perchloride*, inhaled in one grain doses. In hemorrhage from the lungs it was employed in doses of five to seven grains to the ounce. Of all the various preparations of iron, in the numerous diseases of the pharynx, larynx, bronchi and lungs, the *perchloride* has proved the most beneficial.

Several cases of catarrhal irritation of the bronchial tubes of the larynx and trachea, and of excessive secretion from the mucous membrane of the head have been remedied by inhalation of *alum* in doses of twenty to twenty-five grains to the ounce. In some cases more relief has been found from combining the alum with small doses of conium, hyosciamus and opium. Alum, I think, may be usefully employed in more diseases of the respiratory system than any other one remedy. I have used *tannin* very successfully in numerous diseases of these organs, especially in ulceration of the pharynx, larynx, trachea, bronchi and irritation of the mucous membrane of the head. In these astringent remedies I always consider it safer to commence with a small dose; and, if *that* is borne well, then proceed to the larger. I have recently been using several other remedies in these diseases, but have not yet tried them long enough to make a report of their effects, but hope to do so at a future time.

Let me add, in conclusion, that none of these remedies can be relied on alone to cure consumption. They are only *adjuncts. Consumption is a disease of the blood*, and can never be cured by topical remedies alone. As Dr. Rush said, "It is a disease all over." The whole vital fluid is contaminated by the pernicious tubercular condition of "*the blood which is the life of the flesh.*" The whole system must be renovated by good air, gentle out of door exercise, good wholesome food, in as large amount as can be well digested, and a judicious use of such medicines as are calculated to purify and enrich the blood. It is well known that all the fluids and solids of the body can be changed by a proper use of substances taken into the system.

By living properly, consumption may be postponed even in persons predisposed to the disease. When I say, as above, that inhaling remedies will not *alone* cure consumption, I by no means design to discourage this kind of treatment. Its advantages are many, as it conveys the remedy directly to the diseased organs. But, if these could be healed, so long as the blood remained in that condition which first caused the development of tubercles on the lungs, there would remain also the cause for a new development of the disease. The fountain must be made pure, and then we shall have pure blood flowing to every organ.

